# Mixture Buchu and Juniper Compound

**Published:** 1912-01

**Authors:** 


MIXTURE BUCHU AND JUNIPER COMPOUND.—

| | |
|---|---|
| Potassium acetate | 2 oz. |
| Buchu ground (No. 20) | 2 oz. |
| Juniper ground (No. 20) | 1 oz. |
| Syrup (simple) | 5 fl. oz. |
| Oil sassafras | 5 min. |
| Alcohol. | |
| Distilled water | to make 20 fl. oz. |

Moisten the powders with equal parts of alcohol and water, and percolate with 20 per cent alcohol to 14 fluid ounces. Dissolve the oil in 1 fluid ounce of alcohol, add to the previous mixture, and last add the syrup.

Dose: One to two fluid drachms (4 to 8 Cc.).—*Medical Brief.*

---

In any case in which catheterization is required, however careful the nurse or physician, administer hexamethylenamine as a prophylactic against cystitis.—*American Journal of Surgery.*

---

THE *Medical Brief* gives the following as a formula for a compound alum powder useful as a stimulating and healing application for indolent ulcers and to repress fungus granulations:

| | | |
|---|---|---|
| ℞ | Camphor | gr. lx |
| | Phenol | gr. cxx |
| | Dried alum, powdered | ℥vj |

Mix the camphor and phenol in a mortar. When liquified, add the alum, and mix thoroughly.

---

EVEN in the absence of a suggestive history, one should bear in mind the possibility of a foreign body as the cause of localized pain, tenderness, inflammation, or impaired function, when the cause can not otherwise be determined.—*American Journal of Surgery.*

